# Simvastatin Inhibits Glucose Metabolism and Legumain Activity in Human Myotubes

**DOI:** 10.1371/journal.pone.0085721

**Published:** 2014-01-08

**Authors:** Robert Smith, Rigmor Solberg, Linn Løkken Jacobsen, Anette Larsen Voreland, Arild Christian Rustan, G. Hege Thoresen, Harald Thidemann Johansen

**Affiliations:** 1 Department of Pharmaceutical Biosciences, School of Pharmacy, Faculty of Mathematics and Natural Sciences, University of Oslo, Oslo, Norway; 2 Department of Pharmacology, Institute of Clinical Medicine, Faculty of Medicine, University of Oslo and Oslo University Hospital, Oslo, Norway; Stanford University, United States of America

## Abstract

Simvastatin, a HMG-CoA reductase inhibitor, is prescribed worldwide to patients with hypercholesterolemia. Although simvastatin is well tolerated, side effects like myotoxicity are reported. The mechanism for statin-induced myotoxicity is still poorly understood. Reports have suggested impaired mitochondrial dysfunction as a contributor to the observed myotoxicity. In this regard, we wanted to study the effects of simvastatin on glucose metabolism and the activity of legumain, a cysteine protease. Legumain, being the only known asparaginyl endopeptidase, has caspase-like properties and is described to be involved in apoptosis. Recent evidences indicate a regulatory role of both glucose and statins on cysteine proteases in monocytes. Satellite cells were isolated from the *Musculus obliquus internus abdominis* of healthy human donors, proliferated and differentiated into polynuclear myotubes. Simvastatin with or without mevalonolactone, farnesyl pyrophosphate or geranylgeranyl pyrophosphate were introduced on day 5 of differentiation. After 48 h, cells were either harvested for immunoblotting, ELISA, cell viability assay, confocal imaging or enzyme activity analysis, or placed in a fuel handling system with [^14^C]glucose or [^3^H]deoxyglucose for uptake and oxidation studies. A dose-dependent decrease in both glucose uptake and oxidation were observed in mature myotubes after exposure to simvastatin in concentrations not influencing cell viability. In addition, simvastatin caused a decrease in maturation and activity of legumain. Dysregulation of glucose metabolism and decreased legumain activity by simvastatin points out new knowledge about the effects of statins on skeletal muscle, and may contribute to the understanding of the myotoxicity observed by statins.

## Introduction

Simvastatin, a HMG-CoA reductase inhibitor (statin), is prescribed worldwide to patients with hypercholesterolemia to prevent cardiovascular disease and death [1], [2]. Although simvastatin is well-tolerated, side effects like myotoxicity have been reported, ranging from fatigue to life-threatening rhabdomyolysis [3], [4]. Several hypotheses explaining the statin-induced myotoxicity have been put forward, but the underlying mechanism is still poorly understood. The mechanisms for statin-induced myotoxicity are probably multifactorial and at least partly due to a combination of impaired isoprenylation of trafficking proteins [5], [6], altered Ca^2+^ homeostasis [7] and impaired mitochondrial respiratory function [8]–[10]. The mitochondrial respiratory dysfunction observed in statin-treated patients caused glucose intolerance [10]. Also, glucose intolerance was observed in tumor cells after exposure to statin, resulting in decreased glucose uptake and a higher glucose concentration in the conditioned cell medium [11]. Regarding impaired mitochondrial function, many biochemical processes are affected by impaired glucose oxidation and low ATP production, like the activity of the lysosomal H^+^-ATPase, pH of the lysosomes and processing of lysosomal proteases.

Legumain (asparaginyl endopeptidase) is a cysteine protease mainly localized to the lysosomes and was first characterized in mammals in 1997 [12]. Legumain is ubiquitously expressed in mammalian tissue [12], and over-expression is associated with atherosclerotic plaque instability and cancer malignancy [13], [14]. In cancer malignancy legumain has been reported to translocate from the lysosomes to the cell nucleus [15]. Legumain has also been described to participate in apoptosis of *Blastocystis* and neural cells of mice [16], [17]. Furthermore, legumain contributes to the maturation process of cathepsin B and L, two other cysteine proteases [18]. Recently, down-regulation of legumain mRNA in macrophages caused by atorvastatin has been reported as well as decreased cathepsin L activity in statin-treated patients with aortic aneurysms [19], [20]. Legumain cleaves peptide bonds carboxyterminally to asparagine, as well as at aspartate residues at pH below 5 and thus acquiring caspase-like properties [13], [21]. The protease is expressed as a 56 kDa proform, which is autoactivated at acidic pH to 47/46 kDa intermediate forms [22]. The intermediate legumain forms are further enzymatically processed to the mature active 36 kDa form. Also, prolegumain has been reported to be secreted as well as being associated with integrins [13], and can be internalized and subsequently autoactivated [23]. Although the biological and pathological roles of legumain are starting to be elucidated, much is still unknown. Interestingly, increasing the concentration of glucose to the media of human monocytes and murine macrophage-like J774A.1 cells are reported to down-regulate the activity of cathepsin B, D, L and S [24], thus indicating an interesting regulatory role of glucose on lysosomal enzymes.

The overall aim of this study was to investigate effects of simvastatin on glucose metabolism in human myotubes. Skeletal muscles are the major organ for glucose metabolism, and any alteration caused by simvastatin on glucose metabolism in human myotubes could shed light on mechanisms involved in adverse effects and toxicity of statins. Also, the effects of simvastatin on regulation of the cysteine protease legumain were studied in this context.

## Materials and Methods

### Materials

Dulbecco's modified Eagle's medium (DMEM-Glutamax™, 5.5 mM glucose), foetal bovine serum, Ultroser G, penicillin–streptomycin (P/S), amphotericin B, DAPI, XCell SureLock® Mini, NOVEX Tris-Glycine Native Sample Buffer (2X), NOVEX Tris-Glycine Native Running buffer (10X), NuPAGE Bis-Tris 4–12% gels, NuPAGE MOPS SDS running buffer (20X), NuPAGE LDS sample buffer (4X), Alexa®568 donkey anti-mouse (cat. no. A10037), Alexa®488 donkey anti-goat (cat. no. A11055) and ProLong® Gold antifade reagent with DAPI (cat. no. P36935) were obtained from Life Technologies (Paisley, UK). [^14^C-(U)]glucose (107.3 GBq/mmol), [^3^H]deoxyglucose (37 MBq/ml) and [1-^14^C]oleic acid (2 GBq/mmol) were purchased from PerkinElmer NEN® (Boston, MA, USA). Simvastatin was obtained from Toronto Research Chemicals (Ontario, Canada). Insulin Actrapid was from Novo Nordisk (Bagsvaerd, Denmark). Culture plates (6-, 12- and 96-wells) and 25 cm^2^ flasks were obtained from Corning Life-Sciences (Schiphol-Rijk, The Netherlands). OptiPhase Supermix and UniFilter®-96 GF/B were delivered by PerkinElmer (Shelton, CT, USA). CHAPS, DL-dithiotreitol (DTT), trypan blue, triton X-100, mevalonolactone, geranylgeranyl pyrophosphate ammonium salt, farnesyl pyrophosphate ammonium salt, rotenone, oligomycin A, carbonyl cyanide 4-(trifluoromethoxy)-phenylhydrazone (FCCP) and antimycin A were purchased from Sigma-Aldrich (St. Louis, MO, USA). Sodium pyruvate solution, foetal calf serum (FCS) and trypsin-EDTA were purchased from PAA Laboratories GmbH (Pasching, Austria). Nitrocellulose membranes were from Hybond ECL (Amersham Biosciences, Boston, MA, US). Cytoslides (cat. no. 154534), SuperSignal West Dura Extended Duration Substrate and Restore Western Blot Stripping Buffer were purchased from Thermo Fisher Scientific (Rockford, IL, USA). Protein assay reagent, Tween 20, SDS, Precision plus protein standards, goat anti-rabbit IgG HRP-conjugate (cat. no. 170-6515) and goat anti-mouse IgG HRP-conjugate (cat. no. 170-6516) were purchased from BioRad (Copenhagen, Denmark). Goat anti-human legumain (cat. no. AF2199), goat anti-human cathepsin L (cat. no. AF952), goat anti-human cathepsin B (cat. no. AF953) and mouse anti-human arylsulfatase B (cat. no. MAB4415) were purchased from R&D Systems (Abingdon, UK). MitoProfile® Total OXPHOS Human WB Antibody Cocktail (cat. no. ab110411) and rabbit anti-human GLUT1 (cat. no. ab15309) were from Abcam (Cambridge, UK). Rabbit anti-goat IgG HRP-conjugate (cat. no. P0160) was purchased from DAKO (Glostrup, Denmark), whereas mouse anti-human α-tubulin (cat. no. CP06) was obtained from Calbiochem (San Diego, CA, USA). Mouse anti-human GAPDH (cat. no. sc-47724) and mouse anti-human LAMP-2 (sc-18822) were from Santa Cruz (Heidelberg, Germany). Z-Arg-Arg-AMC and Z-Ala-Ala-Asn-AMC were purchased from Bachem (Bubendorf, Switzerland). Non-fat dry milk was from Normilk (Levanger, Norway). Qproteome Cell Compartment Kit was purchased from Qiagen (Hilden, Germany). CellTiter 96® aqueous one solution cell proliferation assay (MTS assay) was obtained from Promega (Madison, Wisconsin, USA). All other chemicals used were standard commercial high-purity quality.

### Ethics Statement

The biopsies were obtained with informed written consent and approval by the Regional Committee for Medical and Health Research Ethics (Oslo, Norway). The research performed in this study was approved, as a part of a larger project, by the Regional Committee for Medical and Health Research Ethics (Oslo, Norway).

### Cell Culturing

Satellite cells were isolated from the *Musculus obliquus internus abdominis* of healthy human donors with no history of statin treatment. The cells were isolated, cultured, proliferated and differentiated as described elsewhere [25]. Briefly, cells were cultured in wells or flasks at a density of approximately 5000–30000 cells/cm^2^ in medium containing DMEM-Glutamax (5.5 mM glucose), 10% FCS, 50 units/ml penicillin/streptomycin (P/S) and 1.25 µg/ml amphotericin B. This medium was substituted after one day with medium containing DMEM-Glutamax, 2% FCS, 2% Ultroser G, 50 units/ml P/S and 1.25 µg/ml amphotericin B and changed every 2–3 days until 80–90% confluence. Myoblast differentiation to myotubes was then induced by changing medium to DMEM-Glutamax with 2% FCS, 34 pM insulin, 50 units/ml P/S and 1.25 µg/ml amphotericin B. The cells were cultured, proliferated and differentiated in humidified 5% CO_2_ atmosphere at 37°C. Incubation with 0–40 µM simvastatin with or without 0.05 or 1 mM mevalonolactone (ML), 3 µM farnesyl pyrophosphate (FPP) or 3 µM geranylgeranyl pyrophosphate (GGPP) in the differentiation medium were introduced on day 5 of differentiation. After 48 h of incubation, cells were either harvested in lysis buffer containing 100 mM sodium citrate, 1 mM disodium-EDTA, 1% n-octyl-β-D-glucopyranoside, pH 5.8 or used for further experiments described later. Cell lysates were freeze-thawed 3 times before analysis by immunoblotting, enzyme activity and total protein measurements. Total protein concentrations were determined by a procedure described elsewhere [26] and standard curves were established using albumin.

### Cysteine Protease Activity Measurements

Legumain activity was measured by recording the cleavage of the peptide substrate Z-Ala-Ala-Asn-AMC. Briefly, 20 µl of cell lysate was added to black 96-well microtiter plates. A kinetic measurement based on increase in fluorescence over 10–60 min was performed after addition of 100 µl legumain assay buffer and 50 µl peptide substrate solution (10 µM Z-Ala-Ala-Asn-AMC) described elsewhere [12], [27].

Cathepsin B activity was measured in a similar way except of using the peptide substrate Z-Arg-Arg-AMC. Briefly, 20 µl of cell lysate was added to black 96-well microtiter plates. Cathepsin B assay buffer [28], [29] and peptide substrate solution (20 µM Z-Arg-Arg-AMC) was added and fluorescence measured. Temperature was kept at 30°C and all measurements were done in triplicate.

Linarites of the assays were established by measuring the initial substrate cleavage rates and limiting the substrate consumption to less than 2% during the measurements. Enzyme activity is presented as unit/mg total proteins (µmol/(min·mg)).

### Immunoblotting

Samples of cell lysate were prepared for NuPAGE electrophoresis according to the manufacturer’s recommendations (Life Technologies). Briefly, samples were mixed with 0.5 M DTT and NuPAGE LDS sample buffer and run along with 5 µl Precision plus protein standard on NuPAGE 4–12% gels in a container with NuPAGE MOPS SDS running buffer. Blotting was performed using 20% methanol, 25 mM Tris, and 0.2 M glycine, pH 8.3. Nitrocellulose membranes were blocked with 5% non-fat milk in Tris-buffered saline containing 0.05% Tween 20 (TBS-T) for 1–2 h at room temperature, and then incubated overnight at 4°C with goat anti-human legumain (1∶1000), goat anti-human cathepsin B (1∶10000), goat anti-human cathepsin L (1∶5000), mouse anti-human GAPDH (1∶10000), mouse anti-human LAMP-2 (1∶500), mouse anti-human α-tubulin (1∶5000), rabbit anti-human GLUT1 (1∶1000), mouse anti-human total OXPHOS cocktail (1∶500) or mouse anti-human arylsulfatase B (ARSB; 1∶500). Further incubation for one hour was performed with appropriate HRP-conjugate of secondary antibodies. After four ten-minute washes in TBS-T, immunoreactive bands on the membranes were detected by SuperSignal West Dura Extended Duration Substrate. Membranes were reprobed after stripping in Restore Western Blot Stripping Buffer as described by the manufacturer (Thermo Fisher Scientific). Immunoband intensities were analyzed by Image 4.0 (BioRad).

### Confocal Imaging

Cells (5×10^4^) were seeded on cytoslides, cultured, differentiated and treated as described above. On day 7 after start of differentiation, cells were fixed with 4% paraformaldehyde in PBS for 10 min on ice and washed twice in PBS before being permeabilized for 5 min with 0.2% Triton X-100. Cells were washed three times with PBS, 0.1% BSA, 0.2% Triton X-100 and 0.05% Tween 20, and then blocked with 10% horse serum for 1 h. Then, immunocytochemical staining was performed using goat anti-human legumain (1∶50) or goat anti-human cathepsin B (1∶100) and mouse anti-human ARSB (1∶50) primary antibodies for 1 h. After three washes, corresponding secondary antibodies were applied (donkey anti-goat; Alexa 488; 1∶250 or donkey anti-mouse; Alexa 568; 1∶500, respectively). The coverslips were mounted in ProLong Gold antifade reagent with DAPI. The cells were observed using a laser-scanning confocal imaging system LSM710 (Carl Zeiss) with equal settings in all experiments.

### ELISA

Established ELISA procedure given by the manufacturer was performed to measure the concentrations of legumain (R&D Systems; MAB21992) in conditioned media from myotubes.

### Cell Viability (MTS)

Cell viability assays were carried out using the manufacturer’s protocol. Briefly, 5×10^4^ cells were cultured, proliferated, differentiated and treated with simvastatin in quadruplicates in 96-wells culture plates. After 24 h, 20 µl of 3-(4,5-dimethylthiazol-2-yl)-5-(3-carboxy-methoxyphenyl)-2-(4-sulfophenyl)-2H-tetrazolium (MTS reagent) were added to each well and incubated for 2 h before absorbance was measured at 490 nm in a microplate reader, Wallac Victor^3^ (PerkinElmer).

### Subcellular Fractionation

Samples of different subcellular fractions were prepared using the Qproteome Cell Compartment Kit according to the manufacturer’s protocol. Purity of fractions was checked with α-tubulin, LAMP-2, ARSB and Lamin B as cytosolic, membrane, soluble membrane and nuclear markers, respectively.

### Uptake and Oxidation of Glucose

Cells were cultured, proliferated and differentiated on 96-well CellBIND® microplates. First, 48 h incubation with simvastatin was started on day 5 of differentiation before analysis with cell-based multiwell assay was performed as described elsewhere [30]. Briefly, medium was removed before addition of [^14^C-(U)]glucose (37 kBq/ml, 0.2 mM) in Dulbeccòs PBS (DPBS) with 10 mM HEPES, containing either 0.1% DMSO, 0.1 µM rotenone, 1 µg/ml oligomycin A, 0.1 µM antimycin A, or 1 µM FCCP. A 96-well UNIFILTER® microplate presoaked with 20 µl 1 M NaOH was mounted on top of the CellBIND® plate, and the cells were incubated at 37°C and 5% CO_2_ for 4 h. The CO_2_ trapped in the filter was then counted by liquid scintillation in a MicroBeta™ Trilux scintillation counter (PerkinElmer). The remaining cell-associated radioactivity was also assessed by liquid scintillation, and the formation of CO_2_ and cell-associated radioactivity was considered as total glucose uptake while the formation of CO_2_ was considered as glucose oxidation.

### Uptake of Deoxyglucose

The cells were cultured, proliferated and differentiated on 12-well plates. First, 5 days after onset of differentiation, treatment with 5 µM simvastatin with or without 1 mM ML were added. After 48 h of incubation, the cells were washed and incubated for 1 h with 140 mM NaCl, 20 mM HEPES, 5 mM KCl, 2.5 mM MgSO_4_ and 1 mM CaCl_2_, pH 7.4, before [^3^H]deoxyglucose (37 kBq/ml, 10 µM) was added and incubated for 15 min. Then cells were washed 3 times with PBS and harvested in 250 µl 0.1 M NaOH. The lysates were counted by liquid scintillation.

### Statistics

The data are represented as mean ± SEM. Student t-test or student paired t-test were performed when appropriate, and statistical significance was considered at p<0.05. All experiments were performed on cells from at least three donors and at least triplicate measurements.

## Results

### Simvastatin Reduced Glucose Uptake and Oxidation in Human Myotubes

**Figure 1:**
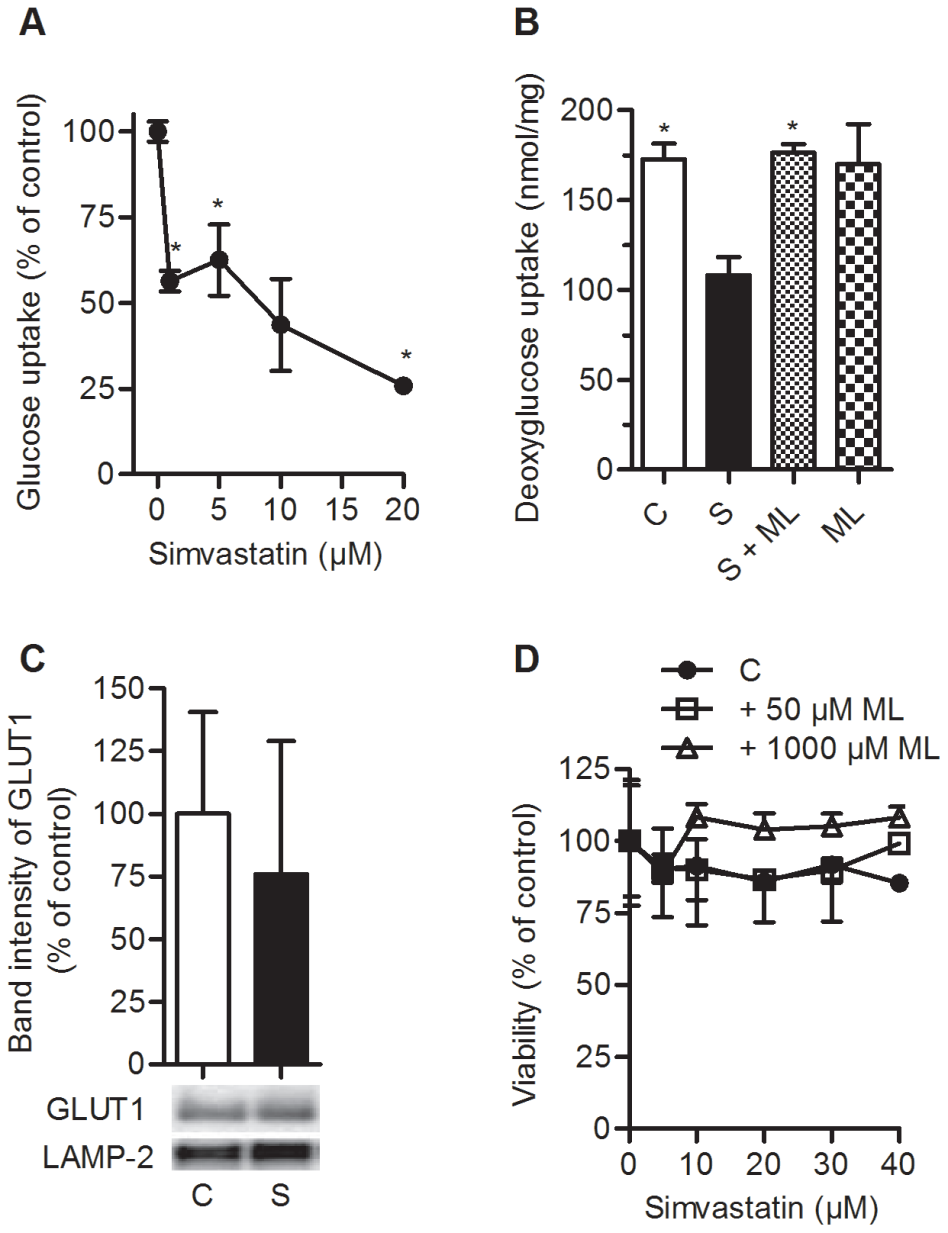
Reduced glucose uptake in myotubes after treatment with simvastatin. **A.** and **B.** Differentiated myotubes were incubated for 48 h with simvastatin (S) with or without mevalonolactone (1 mM; ML) and compared to untreated control (C), prior to incubation with radiolabeled substrates at day 7. **A.** Dose-response of simvastatin on glucose uptake after 4 h incubation with [^14^C(U)]glucose (0.2 mM, 21.5 kBq/ml) using a multiwell trapping device. Radioactivity was measured in cell lysates and in trapped CO_2_ and corrected for total proteins (n = 3–8, student t-test, *p<0.05 vs. untreated). **B.** Effects of 5 µM simvastatin with or without ML on uptake of [^3^H]deoxyglucose (10 µM, 37 kBq/ml, 15 min) (n = 3, student t-test, *p<0.05 vs. S). **C.** Differentiated myotubes were pre-incubated for 48 h with simvastatin (30 µM) before subcellular fractionation. Equal amount of total proteins from the membrane fraction were loaded to the gel and immunoblot analysis performed. LAMP-2 was used as loading control. Quantification of GLUT1 band intensity is shown, corrected for LAMP-2 and normalized to untreated control (C) (n = 4). **D.** Myotubes were treated with 0–40 µM simvastatin with or without 50 µM or 1 mM ML for 48 h before cell viability was analyzed at day 7. The cells were incubated with MTS-reagent for 2 h before absorbance at 490 nm was measured (n = 3, student t-test, *p<0.05 vs. untreated control (C)).

Previous reports have shown that simvastatin reduces glucose metabolism both in cancer cells and adipocytes [11], [31]. In our study using differentiated human myotubes, simvastatin significantly reduced uptake of [^14^C]glucose in a dose-dependent manner with an IC_50_ value of approximately 8 µM (Fig. 1A). The myotubes seemed to be more sensitive to reduced glucose uptake by simvastatin than the embryonic kidney cell line HEK293. Whereas 5 µM simvastatin significantly decreased glucose uptake in the myotubes, no effect was observed in the HEK293 cells (Fig. S1). Reduced glucose uptake caused by 5 µM simvastatin was confirmed using [^3^H]deoxyglucose giving approximately 45% less uptake (Fig. 1B). Treatment with a combination of simvastatin and mevalonolactone (ML; 1 mM) totally prevented the decreased deoxyglucose uptake (Fig. 1B). Since the experiments were done in absence of insulin and GLUT1 is the predominant glucose transporter in human myotubes [32], the GLUT1 expression after exposure to simvastatin was studied. The observed effects of simvastatin were not due to differences in GLUT1 expression (Fig. 1C). To verify that the observed effects of simvastatin were not due to cell death of myotubes, cell viability, total proteins concentrations and caspase-3 expression were studied. There were no differences in cell viability (Fig. 1D), total protein concentrations (Fig. S2A) or immunoband of active caspase-3 (Fig. S2B) after simvastatin treatment.

**Figure 2:**
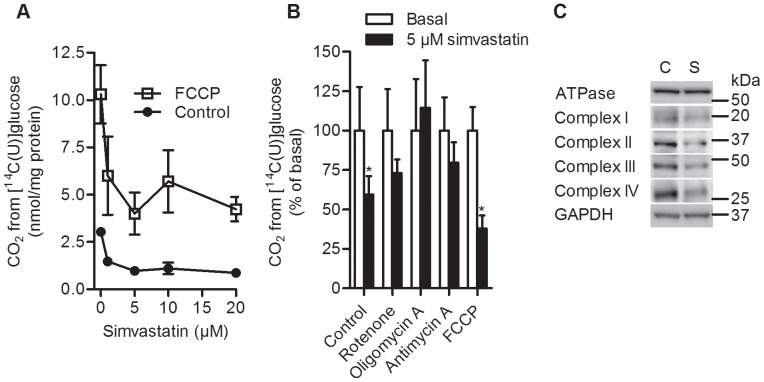
Effects of simvastatin on glucose oxidation and expression of proteins involved in oxidative phosphorylation. **A.** and **B.** Differentiated myotubes were pre-incubated for 48 h with simvastatin (**A**: 0–20 µM; **B**: 5 µM) prior to incubation for 4 h with [^14^C(U)]glucose (0.2 mM, 21.5 kBq/ml) with or without different agents at day 7. **A.** Dose-response of simvastatin on glucose oxidation with or without addition of FCCP (1 µM; n = 3). **B.** Effects on glucose oxidation of 0.1 µM rotenone, 0.1 µM antimycin A, 1 µg/ml oligomycin A or 1 µM FCCP with (black bars) or without (open bars) simvastatin. Radiolabeled [^14^C]CO_2_ was trapped and counted in a MicroBeta® scintillation counter and corrected for total protein (n = 4–8, student paired t-test, *p<0.05 vs. basal). **C.** One representative immunoblot of proteins involved in oxidative phosphorylation from differentiated myotubes incubated for 48 h with (S) or without (C) 10 µM simvastatin is shown. Twenty µg total proteins were loaded to the gel and immunoblot analysis using MitoProfile® Total OXPHOS Human WB Antibody Cocktail and GAPDH were performed (n = 3).

Knowing that the myotubes were fully viable at the simvastatin concentrations used, the observed reduction in glucose uptake could be an indirect effect of impaired oxidation of glucose. Therefore, an uncoupler of oxidative phosphorylation (FCCP) was introduced and shown to increase oxidation of glucose by approximately 3-fold. This reflected a high reserve capacity for glucose oxidation in myotubes, calculated as the difference between oxidation in the presence or absence of FCCP (Fig. 2A). The reserve capacity for glucose oxidation after simvastatin (5 µM) treatment was reduced by approximately 40% (Fig. 2A). To further study the mechanism of simvastatin (5 µM) on oxidative phosphorylation, different inhibitors of the respiratory chain and ATP formation were introduced. Rotenone, oligomycin A, and antimycin A were used to inhibit complex I, ATP synthase and complex III, respectively. No significant effects on glucose oxidation were observed in myotubes treated with any of these agents with or without simvastatin, whereas treatment with FCCP reflected the observation already described above (Fig. 2B). Finally, the effects of simvastatin on the expression of complex I, complex II subunit 30 kDa, complex III core 2, complex IV and ATPase-α-subunit were studied. No significant differences were observed, but there was a tendency of reduced expression of complex I-IV of the respiratory chain by simvastatin (Fig. 2C; Fig. S3).

### Reduced Legumain Activity and Expression in Simvastatin-treated Myotubes

The glucose concentration in the cell culture media has been reported to regulate the activity of cathepsin B, D, L and S in human monocytes and murine macrophage-like J774A.1 cells [24]. Also, atorvastatin has been shown to decrease legumain mRNA in monocytes [20]. Since glucose metabolism was decreased in human myotubes after exposure to simvastatin (Fig. 1, 2), this interesting regulatory role of glucose on lysosomal enzymes led us to investigate whether legumain was affected by simvastatin in human myotubes. Initially, expression and activity of legumain were studied during myotube differentiation and compared to cathepsin B, which is reported to participate in myotube differentiation [33], [34]. Undifferentiated myoblasts (differentiation day 0) showed both legumain and cathepsin B activity at a level of 0.9 (±0.2) and 5.8 (±1.1) µUnit/mg, respectively. Legumain activity was significantly increased both at differentiation day 2 and 5 compared to day 0 (Fig. S4A). Increased activity was due to increased expression of the mature active form (36 kDa) as reflected by immunoblotting (Fig. S4B and C). Cathepsin B activity and expression of the active two-chain form (23 kDa) also showed increasing tendencies throughout myotube differentiation (Fig. S4A, B and D).

**Figure 3:**
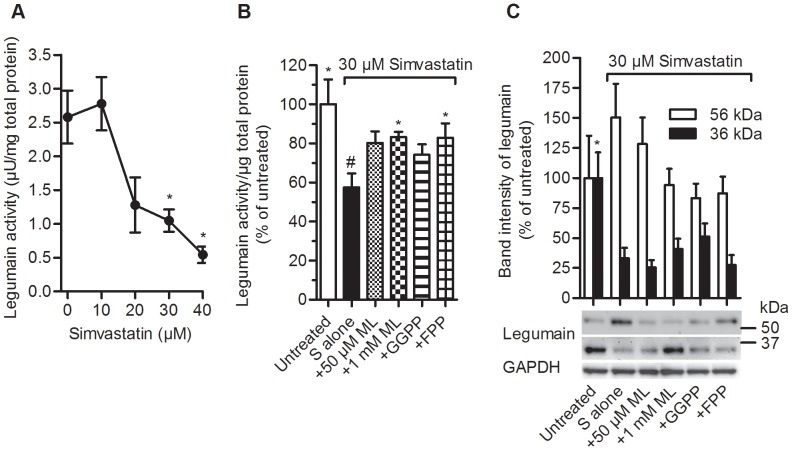
Legumain activity and expression after treatment with simvastatin, mevalonolactone, geranylgeranyl pyrophosphate and/or farnesyl pyrophosphate. Differentiated myotubes were incubated for 48(0–40 µM) with or without mevalonolactone (50 or 1000 µM; ML), geranylgeranyl pyrophosphate (3 µM; GGPP) or farnesyl pyrophosphate (3 µM; FPP) prior to harvesting at day 7. **A.** Dose-dependent effects of simvastatin (0–40 µM) on legumain activity (n = 3–15, student t-test, *p<0.05 vs. 0 µM). **B.** Effects on legumain activity caused by 30 µM simvastatin (S alone) with or without ML, GGPP or FPP. The data are compared and normalized to untreated myotubes (n = 6–12, student t-test, *p<0.05 vs. S alone; n = 12, paired student t-test, #p<0.01 vs. untreated). **C.** Effects on legumain expression caused by 30 µM simvastatin (S alone) with or without ML, GGPP or FPP. Equal amounts of total proteins (10 µg) of cell lysates were separated and immunoblot analyzes were performed. One representative immunoblot is shown and band intensity analysis are normalized to 36 or 56 kDa legumain immunobands, respectively, (n = 4–7, student t-test, *p<0.05 vs. S alone).

Treatment of myotubes on day 5 of differentiation with increasing concentrations of simvastatin for 48 h showed reduced legumain activity in a dose-dependent manner with an IC_50_ value of about 25 µM (Fig. 3A). There was also a tendency of reduced cathepsin B activity caused by increasing simvastatin concentration (data not shown). To study whether inhibition of the HMG-CoA reductase was involved in the reduced legumain activity observed by simvastatin, intermediates of the mevalonate pathway including mevalonolactone (ML), geranylgeranyl pyrophosphate (GGPP) and farnesyl pyrophosphate (FPP) were introduced. The effect of simvastatin (30 µM) on legumain activity was partly prevented by ML (1 mM) or FPP (3 µM; Fig. 3B). Also, the legumain activity measurement was reflected by the expression of the 36 kDa immunoband, representing the mature active form, which was significantly reduced after treatment with simvastatin alone (Fig. 3C, S alone). In addition, a concomitant accumulation of the 56 kDa immunoband was seen, reflecting reduced prolegumain processing. No significant changes were observed by addition of ML, GGPP or FPP (Fig. 3C). Furthermore, fully differentiated myotubes secreted legumain to the conditioned media at a rate of 0.02 (±0.005) pg/cell/day, and the secretion was not affected by treatment with simvastatin (30 µM).

**Figure 4:**
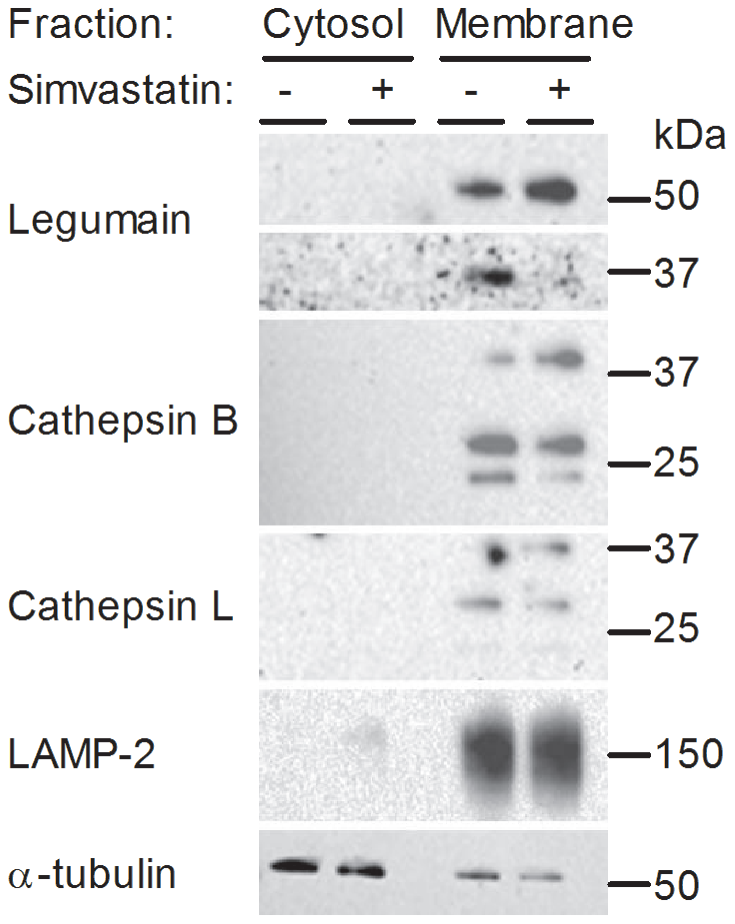
Legumain, cathepsin B and L expressions in cytosolic and membrane fractions of myotubes. Myotubes were treated with (+) or without (−) 30 µM simvastatin for 48 h before subcellular fractionation was performed. One representative immunoblot of legumain, cathepsin B and L in the cytosolic and membrane fractions is shown. All lanes were loaded with equal amount of total proteins and probed with antibodies as indicated. LAMP-2 and α-tubulin are shown as cell compartment controls (n = 3).

To study whether simvastatin could alter the intracellular distribution of lysosomal cysteine proteases, subcellular compartments of myotubes were isolated and analyzed for legumain, cathepsin B and L. Immunoblots showed that both legumain, cathepsin B and L in untreated myotubes were located only in the membrane compartment, comprising cytoplasmic organelles (Fig. 4). After simvastatin treatment, there was no altered subcellular localization of either legumain, cathepsin B or L, as no immunbands were detected in the cytosolic (Fig. 4) or nuclear compartments (data not shown). Subcellular compartment purities were verified by LAMP-2 (membranes), α-tubulin (cytosol) and lamin B (nucleus; not shown), respectively. Simvastatin (30 µM) reduced the expression level of the mature form of legumain (36 kDa) as well as increased the level of prolegumain (56 kDa) in the membrane fraction, confirming the observation in whole myotube lysates (Fig. 3). Also, subcellular presence of legumain and cathepsin B was confirmed by confocal imaging. Legumain seemed to be co-localized in lysosomes with arylsulfatase B (ARSB, a soluble lysosomal enzyme) in untreated myotubes (yellow; Fig. S5A) and treatment with simvastatin did not seem to alter legumain distribution (Fig. S5B). Cathepsin B also seemed to be vesicular although not distinctly co-localized with ARSB (Fig. S5C), and simvastatin caused a more diffuse staining (Fig. S5D).

## Discussion

In this study we observed decreased uptake and oxidation of glucose in human myotubes caused by treatment with simvastatin. Also, legumain (a cysteine protease) was for the first time characterized in human myotubes and decreased legumain activity and expression was observed by simvastatin. The reduction in legumain activity was caused by decreased processing of the 56 kDa prolegumain due to inhibition of the HMG-CoA reductase by simvastatin. These effects of simvastatin on differentiated human myotubes may contribute to the understanding of the pharmacology and toxicology of statins.

The glucose metabolism in human myotubes decreased upon simvastatin treatment, and it is tempting to speculate if this could contribute to hyperglycemia, since some patients taking statins develop decreased insulin sensitivity, insulin resistance and glucose intolerance [10], [35], [36]. Furthermore, the observed decrease in glucose uptake was prevented by concomitant addition of mevalonolactone, suggesting that the mechanism was due to direct inhibition of the HMG-CoA reductase. Although no difference in expression of GLUT1 was observed after simvastatin treatment in this study, regulation of activity or translocation of the GLUT1 transporter or other GLUT transporters could nevertheless account for the effects observed but was not investigated further. Previously, simvastatin has been reported to impair complex I and II in the respiratory chain resulting in ROS accumulation in primary human myotubes established from satellite cells isolated from another source *(Musculus vastus lateralis)* [9]. In our study we detected no differences in CO_2_-production by simvastatin in presence of rotenone (complex I inhibitor), oligomycin A (ATP-synthase inhibitor) or antimycin A (complex III inhibitor). Deviant observations could be due to differences in both origin of myotubes and methods used [9]. Surprisingly, simvastatin reduced FCCP-induced glucose oxidation and oxidative reserve capacity, indicating some effects of simvastatin on cell respiration and mitochondrial function. Therefore, the expressions of proteins in the respiratory chain (complex I-IV and ATP synthase) were studied after treatment with simvastatin. Although no statistically significant changes in the expressions of the analyzed complexes were observed, the expressions tended to decrease and could account for some of the effects.

There may be a link between the two effects observed of simvastatin, impaired glucose metabolism and prolegumain processing, as reduced supply of glucose could cause ATP-depletion in the cell. Decreased ATP levels due to mitochondrial dysfunction have previously been reported in skeletal muscle cells from patients with type 2 diabetes and in rat L6 GLUT4myc myotubes acquiring impaired glucose metabolism [37], [38]. We have recently shown that bafilomycin A1 (a strong inhibitor of the vacuolar type H^+^-ATPase) also reduced the activity of legumain [23]. Since the lysosomal H^+^-ATPase needs ATP to accomplish acidic lysosomal pH [39], our results may indicate that simvastatin could reduce the H^+^-ATPase activity due to possible ATP-depletion resulting in increased lysosomal pH and thus reduced prolegumain processing.

Extracellular glucose is reported to regulate the activity of the cysteine proteases cathepsin B, D, L and S in human monocytes and murine macrophage-like J774A.1 cells [24]. This, together with our observations that simvastatin decreased glucose metabolism in myotubes and the reported down-regulation of legumain mRNA by atorvastatin observed in monocytes [20], made us study the regulatory effects of simvastatin on legumain. We observed a reduced activity of legumain caused by simvastatin and speculated if this could be due to a translocation from the lysosomes to the cytosol since such translocation of legumain has been reported in apoptosis [17]. As a HMG-CoA reductase inhibitor, simvastatin blocks the rate limiting step in the cholesterol synthesis. Cholesterol is a major component of lipid bilayers and cholesterol removal from lysosomal membranes increases the permeability of especially ions and protons, resulting in osmotic imbalance, destabilization and potential leakage of proteins [40], [41]. No lysosomal translocation of legumain was observed using 30 µM simvastatin and could thus not explain the reduced legumain activity observed since the activity is expected to be abolished at neutral cytosolic pH [12]. Cholesterol depletion has also been reported to lead to improper subcellular compartmentalization of phosphoinositides, which are essential for trafficking of intracellular vesicles [42]–[44]. The delocalization of phosphatinositoles is partly due to regulation by cholesterol of the activity of various phosphatinositol kinases at distinct intracellular compartments e.g. type II phosphatidylinositol 4-kinase IIα at the Golgi membrane (PI4KIIα) [45], [46]. Prolegumain needs acidic pH to autoactivate [22]. Therefore, it is possible that some of the effect of simvastatin on inhibition of legumain activity could be due to dysfunctional transport of prolegumain from the Golgi to vesicles with acidic pH, like the late endosomes/lysosomes. Also, depletion of isoprenoids, mainly the geranylgeranyl pyrophosphate (GGPP) and farnesyl pyrophosphate (FPP), have been suggested to be critical for statin-induced myopathy due to their role in prenylation of small GTPases [5], [6], [47]. Inhibition of prenylation will lead to improper intracellular trafficking since functionally small GTPases such as Ras and Rab are essential for targeting, tethering, uncoating and formation of intracellular vesicles [5], [47], [48]. GGPP has been shown to prevent the decreased prenylation of Rab1 GTPase by fluvastatin [6]. Here, down-regulation of legumain activity by simvastatin was significantly prevented by FPP or mevalonolactone (ML), but not by GGPP, indicating that inhibition of prenylation of Rab1 GTPase may not account for the effects observed.

The activity of cysteine proteases are strictly controlled within a cell, and uncontrolled legumain activity is associated with serious diseases like atherosclerotic plaque instability and malignant cancer [13], [14]. A pro-survival role of legumain has been reported in the parasite *Blastocystis* of which inhibition of legumain activity is associated with increased programmed cell death [16]. In this study legumain activity and expression were decreased by simvastatin, but not cell viability, cell total protein content or caspase-3 expression indicating that proteases other than legumain are involved in statin-induced cell death. Down-regulation of legumain could still contribute to a general distortion of protease/kinase activity possibly leading to toxicity, but this needs to be further investigated.

Simvastatin was introduced 5 days into myotube differentiation to mimic an *in vivo* condition since reports have demonstrated that toxic effects of statins mainly affect differentiated myotubes and not myoblasts [6], [9]. Simvastatin was used in our study since this statin has the highest frequency of reported myotoxic effects [49], and the lactone forms of statins are more potent in causing myotoxicity [50]. The concentration of simvastatin used in present study is in the micromolar range, but the achieved plasma concentration after administration of a clinical simvastatin dose is in the nanomolar range. Still, the statin concentrations used in this study are in range with other published *in vitro* experiments [50]. Since the exposure of simvastatin *in vitro* must be for shorter periods of time (days) compared to long term therapeutic *in vivo* use, and the primary cell culture has limited life span, it can be argued that higher statin concentrations are needed to detect cellular responses. Nevertheless, in our study and as mentioned above, the simvastatin concentrations used in this study (5–40 µM) caused no significantly effects on cell viability, cell total protein contents or expression of active caspase-3. In contrast, it has previously been reported reduced myotube viability by 5 µM simvastatin [9], [51].

In conclusion, this study shows that simvastatin reduced both glucose metabolism and legumain activity in myotubes. Both phenomenon are of importance to fully understand the pharmacology and toxicology of statin treatment and needs to be further investigated.

## Supporting Information

Figure S1 **Simvastatin reduced glucose uptake in myotubes but not in HEK293 cells.** Differentiated myotubes or HEK293 cells were incubated for 48 h with or without 5 µM simvastatin prior to incubation for 4 h with [^14^C(U)]glucose (0.2 mM, 21.5 kBq/ml) using a multiwell trapping device. Radioactivity was measured in cell lysates and corrected for total proteins (n = 4–8, student t-test, *p<0.05 vs. untreated).
(TIF)

Figure S2 **Effects of simvastatin on total protein content (A) and caspase-3 expression (B) in differentiated myotubes.** Differentiated myotubes were incubated for 48 h with or without simvastatin (5–40 µM). **A.** Total protein concentrations in cell lysates were measured and normalized to untreated control (n = 17). **B.** Equal amounts of total proteins (10 µg) of cell lysates were analyzed for caspase-3 by immunoblotting. One representative immunoblot is shown and band intensity analysis of procaspase-3 are normalized to untreated control (n = 3).
(TIF)

Figure S3 **Expressions of ATPase, complex I, II, III and IV after simvastatin treatment.** Differentiated myotubes were pre-incubated for 48 h with or without (control) simvastatin (10 or 30 µM; pooled results) prior to harvesting at day 7. Ten-twenty µg total proteins were loaded per well and immunoblotting using MitoProfile® Total OXPHOS Human WB Antibody Cocktail was performed. GAPDH was used as loading control. Quantification of immunobands are shown, corrected for GAPDH and normalized to control (n = 7).
(TIF)

Figure S4 **Characterization of legumain and cathepsin B during differentiation of human myotubes.** Myoblasts (50,000 cells/well) were cultured and proliferated to 80–90% confluence before start of differentiation (day 0), and cells were harvested at day 0, 2, 5 and 7. **A**. Legumain and cathepsin B activities in cell lysates were measured by cleavage of fluorogenic peptide substrates (n = 3–4, student t-test, *p<0.05 vs. differentiation day 0). **B.** Representative immunoblots of cell lysates are shown. Equal amounts of total proteins (10 µg/well) were applied to the gel and immunoblot analysis was performed. Immunoband intensities of pro- and active forms of legumain (**C**) and cathepsin B (**D**) were measured and normalized to differentiation day 0 (n = 3).
(TIF)

Figure S5 **Localization of legumain and cathepsin B in myotubes treated with or without simvastatin.** Myotubes were cultured, differentiated, and incubated for 48 h without (control) or with 30 µM simvastatin prior to fixation at day 7. After fixation, the cells were permeabilized, blocked and further incubated with primary antibodies against legumain (green; A and B) or cathepsin B (green; C and D) and arylsulfatase B (ARSB; red). Secondary antibodies against the species of the primary antibodies and DAPI (blue) were used. The cells were photographed with identical camera and laser settings by LSM710 confocal microscopy (scale bars, 20 µm).
(TIF)
