# Correction: Simvastatin Inhibits Glucose Metabolism and Legumain Activity in Human Myotubes

**DOI:** 10.1371/journal.pone.0093202

**Published:** 2014-03-20

**Authors:** The PLOS ONE Staff

The affiliation for the seventh author is incorrect. Harald Thidemann Johansen is not affiliated with #2 but with #1 Department of Pharmaceutical Bioscience, School of Pharmacy, Faculty of Mathematics and Natural Sciences, University of Oslo.
