# Supplementary figures and images for: Simvastatin Inhibits Glucose Metabolism and Legumain Activity in Human Myotubes
Source: PLoS One. 2014 Jan 8;9(1):e85721. doi: 10.1371/journal.pone.0085721 (PMC3885717; doi:10.1371/journal.pone.0085721)

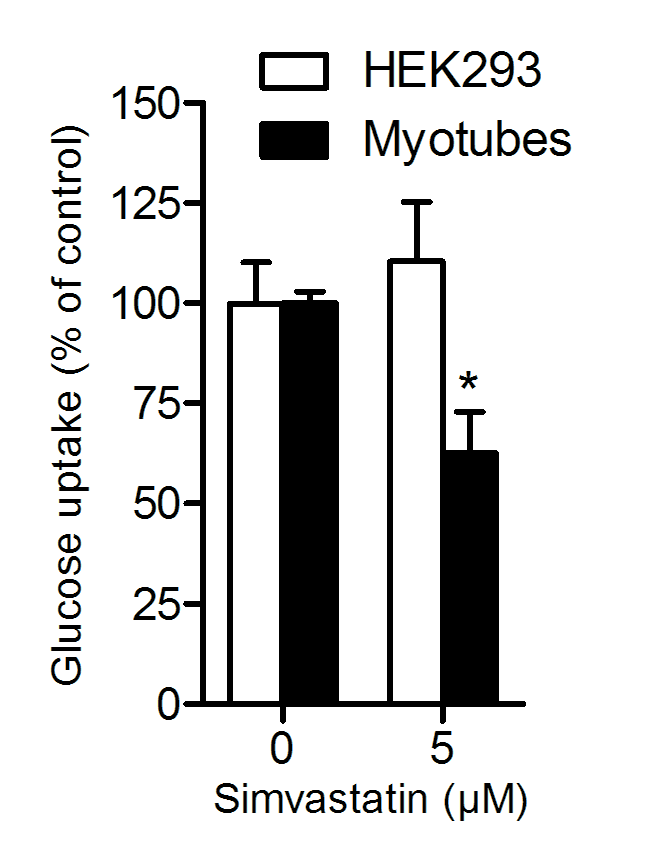

Supplement: Figure S1 — Simvastatin reduced glucose uptake in myotubes but not in HEK293 cells. Differentiated myotubes or HEK293 cells were incubated for 48 h with or without 5 µM simvastatin prior to incubation for 4 h with [14C(U)]glucose (0.2 mM, 21.5 kBq/ml) using a multiwell trapping device. Radioactivity was measured in cell lysates and corrected for total proteins (n = 4–8, student t-test, *p<0.05 vs. untreated). (TIF) [file pone.0085721.s001.tif]

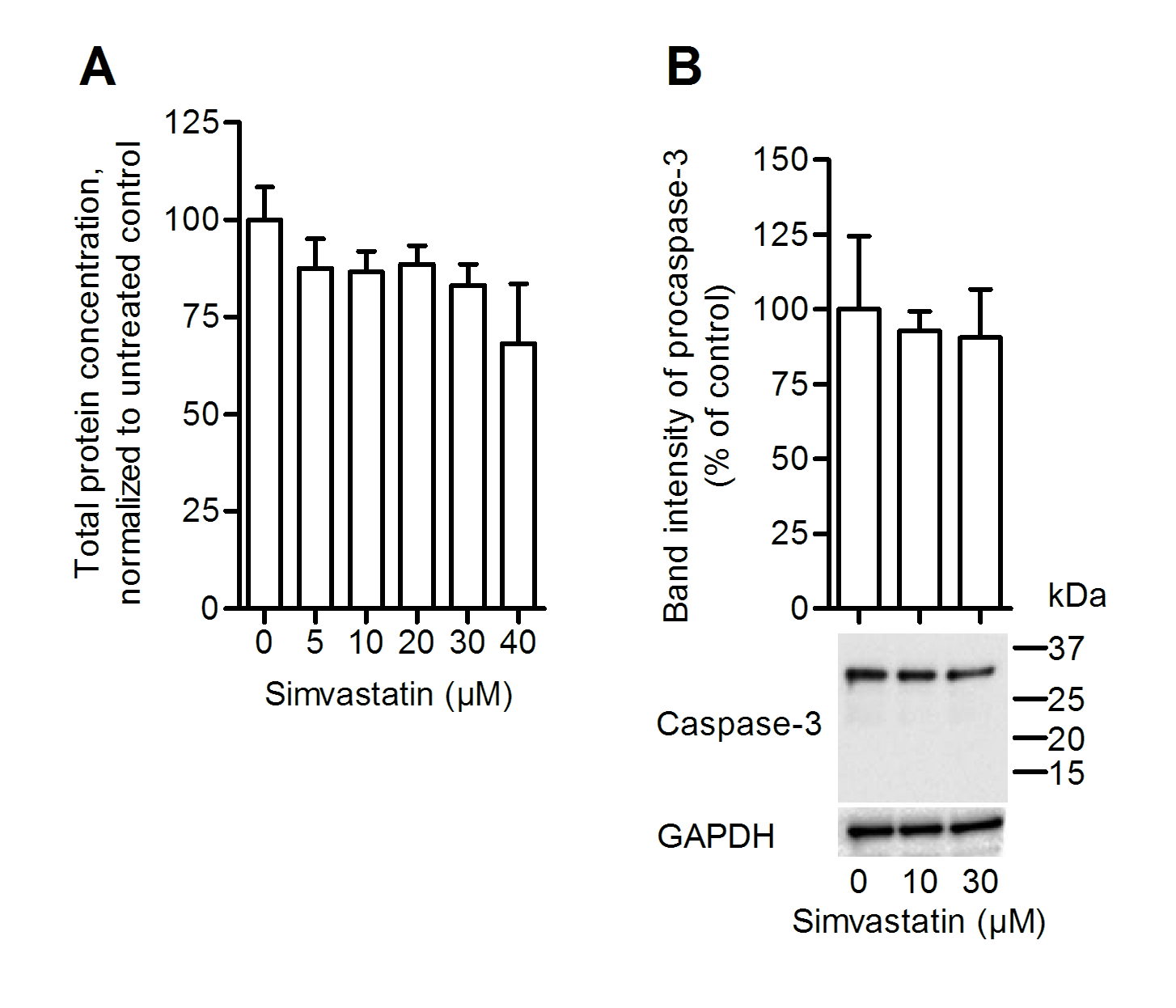

Supplement: Figure S2 — Effects of simvastatin on total protein content (A) and caspase-3 expression (B) in differentiated myotubes. Differentiated myotubes were incubated for 48 h with or without simvastatin (5–40 µM). A. Total protein concentrations in cell lysates were measured and normalized to untreated control (n = 17). B. Equal amounts of total proteins (10 µg) of cell lysates were analyzed for caspase-3 by immunoblotting. One representative immunoblot is shown and band intensity analysis of procaspase-3 are normalized to untreated control (n = 3). (TIF) [file pone.0085721.s002.tif]

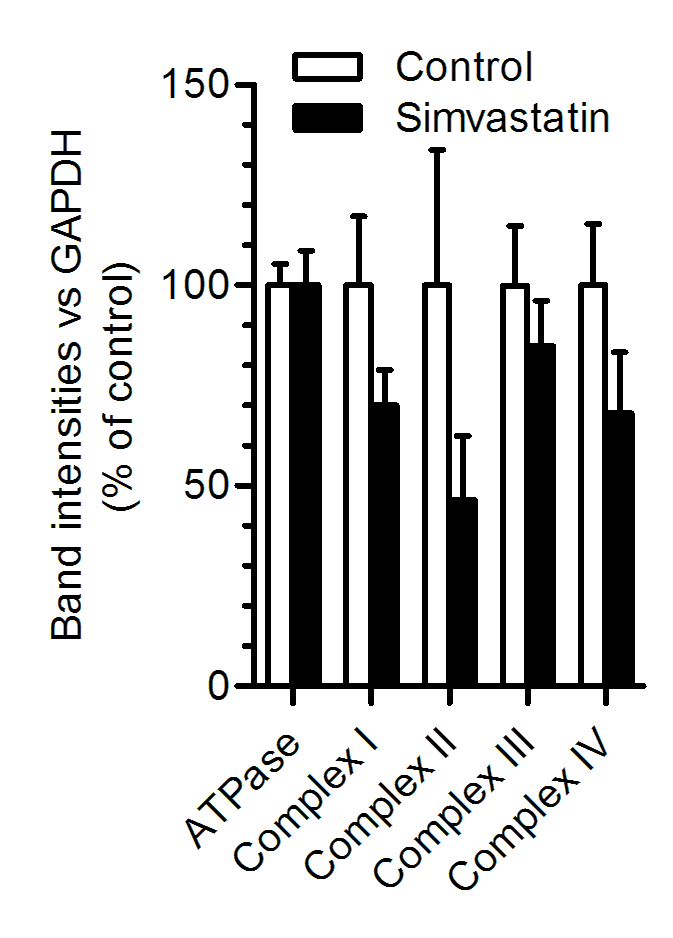

Supplement: Figure S3 — Expressions of ATPase, complex I, II, III and IV after simvastatin treatment. Differentiated myotubes were pre-incubated for 48 h with or without (control) simvastatin (10 or 30 µM; pooled results) prior to harvesting at day 7. Ten-twenty µg total proteins were loaded per well and immunoblotting using MitoProfile® Total OXPHOS Human WB Antibody Cocktail was performed. GAPDH was used as loading control. Quantification of immunobands are shown, corrected for GAPDH and normalized to control (n = 7). (TIF) [file pone.0085721.s003.tif]

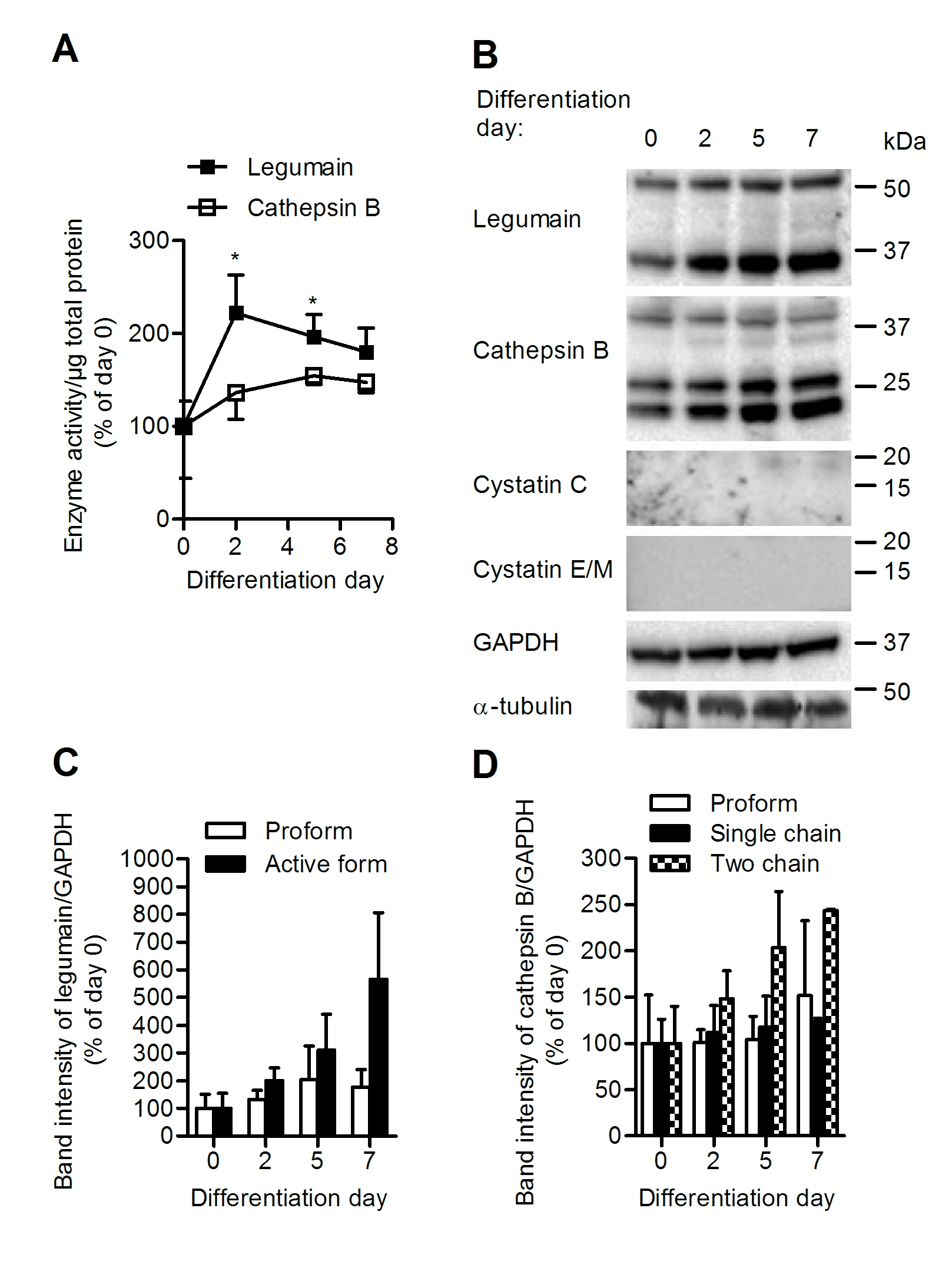

Supplement: Figure S4 — Characterization of legumain and cathepsin B during differentiation of human myotubes. Myoblasts (50,000 cells/well) were cultured and proliferated to 80–90% confluence before start of differentiation (day 0), and cells were harvested at day 0, 2, 5 and 7. A. Legumain and cathepsin B activities in cell lysates were measured by cleavage of fluorogenic peptide substrates (n = 3–4, student t-test, *p<0.05 vs. differentiation day 0). B. Representative immunoblots of cell lysates are shown. Equal amounts of total proteins (10 µg/well) were applied to the gel and immunoblot analysis was performed. Immunoband intensities of pro- and active forms of legumain (C) and cathepsin B (D) were measured and normalized to differentiation day 0 (n = 3). (TIF) [file pone.0085721.s004.tif]

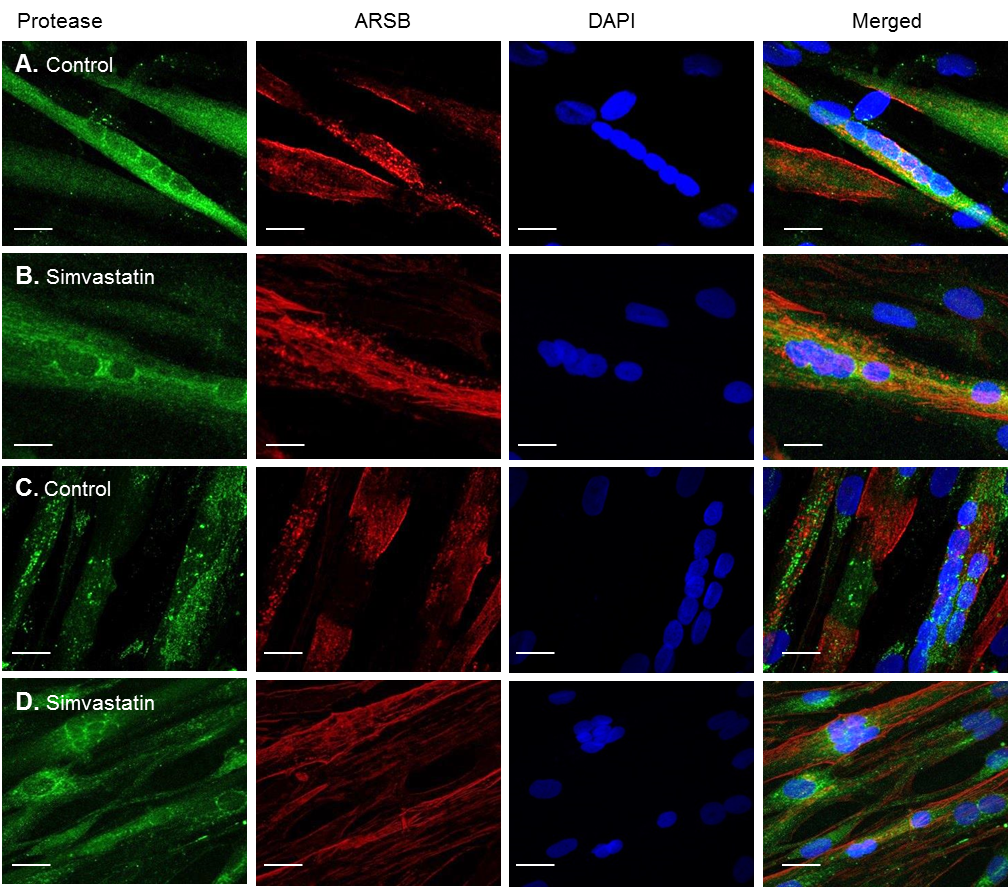

Supplement: Figure S5 — Localization of legumain and cathepsin B in myotubes treated with or without simvastatin. Myotubes were cultured, differentiated, and incubated for 48 h without (control) or with 30 µM simvastatin prior to fixation at day 7. After fixation, the cells were permeabilized, blocked and further incubated with primary antibodies against legumain (green; A and B) or cathepsin B (green; C and D) and arylsulfatase B (ARSB; red). Secondary antibodies against the species of the primary antibodies and DAPI (blue) were used. The cells were photographed with identical camera and laser settings by LSM710 confocal microscopy (scale bars, 20 µm). (TIF) [file pone.0085721.s005.tif]
